# What Are the Game Changers in Total Knee Arthroplasty? A Narrative Review

**DOI:** 10.3390/jpm15080389

**Published:** 2025-08-20

**Authors:** Andrea Baldini, Damiano Ardiri, Lorenzo Benvenuti, Mattia Chirico, Enrico Fiorilli, Alessandro Singlitico, Filippo Leggieri

**Affiliations:** 1 Orthopaedic Unit, Istituto Fiorentino di Cura e Assistenza (IFCA), 50139 Florence, Italy; drbaldiniandrea@yahoo.it; 2 BIOMORF Department of Biomedical, Dental, Morphological and Functional Images, University of Messina, 98100 Messina, Italy; damianoardiri7@gmail.com; 3 Department of Orthopaedic and Trauma Surgery, A.O.U. Policlinico “G. Martino”—Via Consolare Valeria 1, 98124 Messina, Italy; 4 1st Orthopaedic and Traumatologic Clinic, IRCCS Istituto Ortopedico Rizzoli, Via G.C. Pupilli 1, 40136 Bologna, Italy; lorenzo.benvenuti@ior.it; 5 Department of Clinical Orthopaedic, University of Florence, A.O.U. Careggi CTO—Largo Palagi 1, 50139 Florence, Italy; 6 Ortopedia e Traumatologia, Università degli Studi di Torino, 10126 Torino, Italy; enrico.fiorilli@unito.it; 7 Adult Reconstruction and Joint Replacement Service, Division of Orthopaedics and Traumatology, Fondazione Policlinico Universitario Agostino Gemelli IRCCS, 00186 Roma, Italy

**Keywords:** arthroplasty, replacement, knee, cryotherapy, treatment outcome, patient selection, surgical procedures, operative, cost–benefit analysis

## Abstract

**Background**: Total knee arthroplasty (TKA) has evolved significantly, yet achieving consistently optimal outcomes remains challenging across diverse patient populations. This comprehensive narrative review identifies evidence-based “game changers” that genuinely transform TKA success while distinguishing them from interventions lacking clinical superiority. The analysis organizes findings across three perioperative phases: preoperative optimization, intraoperative techniques, and postoperative management. Preoperative game changers include end-stage bone-on-bone osteoarthritis, preoperative medical optimization of patients performed by dedicated practitioners, cryocompression therapy, and perioperative dexamethasone administration. Intraoperative interventions demonstrating substantial impact encompass reduced surgical time and optimized surgical instrumentation, personalized alignment, medial congruent bearings, cementless implants for high-demanding and high-BMI patients, and perioperative tranexamic acid. Postoperative game changers include early mobilization following surgery, venous thrombo-embolic prophylaxis avoiding high-bleeding-risk pharmaceuticals, and multimodal pain management. The review also identifies those initial promises without established clinical advantages, or “fake game changers”, that consume resources without meaningful benefits. This evidence synthesis demonstrates that TKA optimization requires systematic implementation of validated interventions rather than pursuing technological innovations indiscriminately. The future of TKA lies in evidence-based adoption of proven strategies that translate to genuine patient outcome improvements rather than merely increasing procedural complexity.

## 1. Introduction

Total knee arthroplasty (TKA), while generally successful, still presents certain challenges for orthopedic surgeons in consistently delivering the optimal pain relief and functional improvement that patients seek from this surgery [1]. Despite notable advances in surgical methods and implant designs, achieving uniformly predictable results can be difficult across all patient populations [2].

The adult knee reconstruction surgeons actively seek breakthrough interventions, or game changers, that could revolutionize TKA patient outcomes. In TKA surgery, identifying such transformative approaches means separating genuinely beneficial innovations from those that initially appear promising but ultimately fail to provide lasting clinical improvements.

The purpose of this narrative review was to identify evidence-based “game changers” in total knee arthroplasty—interventions that have genuinely transformed patient outcomes and altered clinical practice—while distinguishing them from practices that lack demonstrated clinical superiority. We aimed to provide clinicians with a practical framework for implementing interventions with proven benefits across the perioperative journey, from preoperative preparation through postoperative recovery.

This review employed a narrative approach to identify interventions that have demonstrated a transformative impact in total knee arthroplasty. The review process leveraged the authors’ clinical experience in adult knee reconstruction surgery to identify key intervention domains and select high-impact evidence through purposeful sampling. Studies were prioritized based on their demonstrated ability to change clinical practice, focusing on landmark randomized controlled trials, systematic reviews and meta-analyses, large-scale registry studies, and consensus statements from major organizations. This approach enabled a comprehensive synthesis of clinically relevant evidence while focusing specifically on interventions that have proven transformative in real-world practice settings.

## 2. Preoperative Stage

### 2.1. Bone-on-Bone Osteoarthritis

Patient selection serves as a key factor in TKA success, with the primary goal being the achievement of the Patient Acceptable Symptom State (PASS) [3]. Evidence indicates that patients with advanced Kellgren–Lawrence (KL) Grade 4 bone-on-bone osteoarthritis tend to experience better outcomes compared to those with early-stage disease, who may face suboptimal results more frequently [4,5]. Notably, 50% of patients presenting with unexplained painful TKAs lacking identifiable pathology had preoperative early-stage osteoarthritis (KL 1-2), compared to only 5–10% in control populations [4]. This corresponds to a 4.2-fold increased risk of postoperative dissatisfaction and unexplained pain among patients with mild radiographic changes [5]. From a functional perspective, patients with severe compartmental disease show greater improvements, with knee scores surpassing those of patients with mild disease by 8 and 5 points for medial and lateral disease, respectively [6]. Furthermore, KL Grade 4 patients achieve notably less pain at 12 months compared to lower grades [7]. Performing TKA on patients with early-stage osteoarthritis can lead to challenging outcomes—when a knee without severe structural damage receives an implant, patients may experience ongoing awareness of their artificial joint that could have been avoided. This evidence suggests that careful patient selection based on preoperative disease severity represents a crucial element in TKA outcomes, emphasizing the importance of following evidence-based selection criteria to enhance surgical success.

### 2.2. Patient Medical Optimization

Comprehensive preoperative medical optimization has become an important intervention that can significantly improve surgical outcomes, representing a shift from traditional practices of accepting patients in their current medical state to evidence-based systematic optimization. Current data show that patients presenting with moderate to severe anemia experience notably higher complication rates, with hemoglobin levels below 11.0 g/dL associated with a five-fold increased risk compared to optimized patients [8]. Similarly, poorly controlled diabetes presents considerable perioperative challenges, with elevated glucose levels linked to increased rates of periprosthetic joint infection, readmission, and reoperation [9,10]. The implementation of structured optimization protocols, such as the Perioperative Orthopaedic Surgical Home (POSH) initiative, has produced positive results across multiple outcome measures [10]. Optimized patients show reduced readmission rates at both 30 and 90 days, decreased discharge to post-acute care facilities, and lower healthcare costs, with non-optimized patients experiencing episode-of-care costs that are 15–33% higher depending on baseline risk profiles [11]. The economic benefits extend beyond immediate perioperative savings, with optimization protocols reducing overall arthroplasty costs by up to 7.6% while improving clinical outcomes [12]. Specialized geriatric consultation can further enhance this optimization approach, with preoperative geriatrician involvement helping to reduce emergency department visits and facilitate higher rates of home discharge [13]. This evidence suggests that preoperative medical optimization represents a valuable component of contemporary arthroplasty care, offering a clinically beneficial intervention that can enhance both patient outcomes and healthcare efficiency.

### 2.3. Cryotherapy

Cryotherapy has established itself as a beneficial intervention in TKA care, supported by substantial evidence demonstrating its effectiveness through various physiological mechanisms [14]. Cold exposure induces vasoconstriction, which helps reduce local metabolism and inflammation while decreasing capillary permeability and edema formation. Additionally, it activates pain control pathways and creates conditions that may inhibit bacterial growth [15]. When combined with compression, therapeutic cold can penetrate more effectively into deeper tissues while helping prevent fluid accumulation through reduced vascular-tissue pressure gradients.

The cooling and compression effects influence joint temperatures, which correlate with lower prostaglandin E2 levels, thereby reducing synovial inflammation and decreasing key inflammatory mediators, including interleukin-6, interleukin-1 beta, and vascular endothelial growth factor [16]. These physiological effects contribute to improved achievement of rehabilitation milestones and measurable clinical benefits, with patients experiencing reduced pain scores and decreased opioid consumption during recovery [17]. Recent developments in preoperative cryocompression application have shown promising potential for enhancing surgical outcomes beyond traditional postoperative protocols. A notable randomized controlled trial examining intensive preoperative cryocompression applied for 1.5 h before surgery found that this intervention reduced bone temperature after arthrotomy to 21 °C, resulting in lower early postoperative inflammatory markers, shorter time to achieve rehabilitation milestones, and improved flexion range of motion at 20 postoperative days compared to patients who received only postoperative cryocompression therapy [18].

### 2.4. Intravenous High-Dose Corticosteroids

Perioperative corticosteroid administration has evolved from historical concerns about immunosuppression and infection risk to evidence-based recognition as a valuable intervention in arthroplasty outcomes. Evidence has shown that dexamethasone administration, especially at higher doses of 24 mg with potential repeat dosing on postoperative days 1–2, can improve multiple patient parameters [19]. The physiological mechanisms underlying corticosteroid effectiveness involve suppression of the inflammatory cascade with reductions in interleukin-6, C-reactive protein, and other inflammatory markers that typically increase following surgical trauma. Clinical benefits appear across various domains, with patients experiencing reductions in postoperative nausea and vomiting, decreased pain scores both at rest and during activity, reduced opioid consumption, and enhanced functional recovery, including improved ambulation distances and earlier achievement of rehabilitation milestones [20]. Large-scale randomized controlled trials have shown that high-dose systemic corticosteroids provide better outcomes compared to low-dose protocols, with two-dose regimens offering additional benefits over single-dose administration [21,22]. Meta-analyses encompassing over 1000 patients demonstrate improvements in pain management, reduced inflammatory marker levels, decreased antiemetic requirements, and enhanced early rehabilitation without increased risks of deep infection, gastrointestinal complications, or other serious adverse events [23]. Additionally, corticosteroid administration has been associated with reduced endothelial damage markers, suggesting potential protective effects on vascular integrity during the perioperative period [24]. The evidence supports perioperative corticosteroids as a beneficial component of contemporary enhanced recovery protocols, offering improvements in patient experience, functional outcomes, and overall surgical success while maintaining a favorable safety profile when appropriately dosed and administered.

## 3. Intraoperative Stage

### 3.1. Personalized Alignment

The debate surrounding personalized surgical technique versus standardized mechanical alignment continues to evolve. The Coronal Plane Alignment of the Knee (CPAK) classification system has identified distinct patterns of responsiveness, with types 1 and 4 appearing to be suitable candidates for personalized alignment strategies. In these populations, kinematic alignment may achieve better outcomes [25]. This technical approach becomes particularly relevant when considering that surgeon skill and joint receptivity to complex ligamentous balancing procedures vary considerably, which can create variable outcomes with mechanical alignment approaches [2]. Mechanical alignment in these morphotypes creates trapezoidal resection gaps requiring extensive soft tissue releases that depend on individual surgeon expertise and tissue response. In contrast, maintaining cuts that preserve existing kinematics through alignment closer to patient morphotype can result in balanced knees, potentially reducing the need for complex balancing procedures and their associated uncertainties [26]. This technical approach may translate to improved clinical results, as patients can avoid potential complications of extensive soft tissue manipulation.

CPAK type 2 patients, despite representing neutral constitutional alignment, may still benefit from personalized approaches, suggesting that respecting the native joint line orientation can provide clinical advantages even in seemingly neutral anatomy [27]. In cases presenting with neutral alignment patterns, specifically CPAK type 5 patients, personalized approaches may not differ significantly from mechanical alignment approaches. Evidence remains limited for valgus constitutional patterns and severe constitutional deformities in CPAK types 1 and 4, where extensive anatomical distortion may exceed the compensatory capacity of alignment-preserving techniques.

### 3.2. Tranexamic Acid and Watertight Closure

Tranexamic acid and watertight closure represent valuable interventions that can enhance perioperative bleeding management and wound healing outcomes in TKA. Tranexamic acid has evolved beyond its traditional role as a systemic antifibrinolytic agent to become a therapeutic intervention with evidence supporting preoperative administration of at least 1 g, potential postoperative doubling, and intra-articular application that has gained evidence-based support alongside oral routes [28]. The intra-articular application of 30–40 mL tranexamic acid serves dual purposes: achieving effective local hemostasis and providing a method for testing watertight capsular closure integrity, helping ensure that subsequent accelerated rehabilitation protocols do not compromise wound healing [29]. This approach enables sequential suturing from capsule to subcuticular and intradermal layers, preventing articular fluid leakage during early mobilization that could compromise healing or increase infection risk [30]. The maintenance of knee flexion postoperatively for several hours promotes sustained articular hemostasis and prevents hemarthrosis formation, directly improving clinical outcomes through reduced pain, swelling, and functional limitations [31]. Contemporary wound closure techniques have evolved to incorporate advanced materials and methods that optimize both efficiency and outcomes [32]. Barbed sutures demonstrate strong expert consensus support, with widespread agreement for reducing operative time and resource consumption, along with support for reducing complications and improving cosmetic results [33]. Meta-analyses encompassing hundreds of patients show closure time reductions averaging 4–7 min, lower wound complication rates, reduced overall costs despite higher material expenses, and shorter hospital stays with higher rates of early discharge [32,34].

### 3.3. Cementless Fixation in Young or Obese Patients

The shift toward cementless components represents a notable development in contemporary TKA, supported by evidence demonstrating favorable performance in specific patient populations and advancing implant technologies. American registry data shows steady annual increases in cementless utilization, reaching 22% in 2023, with continued growth anticipated as clinical evidence develops [35]. This trend reflects growing recognition that cemented fixation may face challenges in high-demand cohorts, specifically young, active patients, males, and obese individuals, where mechanical stresses may exceed the capacity of cement-bone interfaces.

Registry analysis indicates increased loosening rates in males under 65 years with cemented versus cementless prostheses, while patients with BMI exceeding 35–40 show higher failure rates with cemented implants compared to biological fixation alternatives [35,36]. The improved performance of cementless prostheses has been facilitated by advanced implant designs incorporating four-peg and keel configurations that achieve reliable primary stability, addressing previous concerns about initial fixation adequacy [37]. Meta-analyses in younger patients demonstrate benefits favoring cementless over cemented fixation across multiple domains, including improved patient-reported outcome measures, reduced pain scores, enhanced range of motion, and lower rates of component radiolucency [37,38]. In obese populations, the evidence appears particularly supportive, with cementless fixation demonstrating aseptic loosening rates of only 0.9% compared to 18.8% in cemented cohorts, translating to survivorship rates of 99.1% versus 88.2% at eight years [39]. Pooled analyses suggest benefits of cementless implants in high BMI patients, with odds ratios of 0.17 for all-cause revisions and 0.15 for aseptic loosening, representing substantial risk reductions that support the growing use of biological fixation [40]. This evidence suggests that cementless fixation may be a preferred approach for younger, more active, and heavier patients where mechanical demands may exceed the long-term capacity of cemented interfaces, representing an evolution in fixation philosophy that can optimize outcomes for patients most likely to outlive their implants.

### 3.4. Anatomical Local Infiltration Anesthesia and “Curved” Anterolateral Skin Incision

Anatomical Local Infiltration Anesthesia (LIA) and strategic anterolateral skin incision represent refined intraoperative techniques that address the complex neuroanatomy surrounding the knee to enhance postoperative comfort and functional outcomes [41,42]. Anatomical LIA improves upon traditional infiltration techniques by targeting specific nerve structures rather than employing generalized tissue flooding, with key targets including the genicular femoral and tibial nerves, particularly focusing on the saphenous nerve complex [43,44]. Contemporary understanding of saphenous nerve anatomy has enabled the development of reproducible intraoperative techniques that can achieve effective neural blockade without requiring specialized ultrasound guidance or additional procedural time.

The approach involves accessing the nerve approximately 8 cm deep at a 30° inclination parallel to the bone surface, using the femoral artery as a reliable anatomical landmark confirmed by aspiration bleeding, enabling effective adductor canal blockade that can rival formal regional anesthetic techniques while maintaining surgical workflow efficiency. This precision targeting may achieve improved pain control compared to traditional infiltration methods while minimizing the systemic absorption and potential toxicity associated with large-volume local anesthetic administration.

The complementary anterolateral skin incision, while not universally applied, may provide benefits for specific patient populations, including those requiring kneeling ability, patients with hypertrophic subcutaneous tissue in the saphenous territory, women, and individuals at high risk for postoperative pain syndromes [45,46]. This incision runs lateral to the extensor mechanism, followed by standard anteromedial capsulotomy, strategically avoiding saphenous nerve branches that can cause persistent anterior knee discomfort and functional limitations.

Patients with healed anterolateral incisions often experience reduced anterior sensitivity issues, particularly beneficial for kneeling, exercise, and ground contact activities that may be compromised with traditional medial approaches [45,46]. Additionally, this technique may prevent autonomic denervation reactions that manifest as unexplained lateral herpetic-like skin changes, which represent saphenous nerve denervation responses rather than infectious processes [47]. The integration of anatomical LIA with strategic incision placement represents an understanding of knee neuroanatomy that may translate to improved patient comfort, enhanced functional recovery, and reduced chronic pain syndromes that can affect long-term satisfaction with TKA outcomes.

### 3.5. Reduced Surgical Time

Reduced surgical time represents an important factor in arthroplasty outcomes, with procedures completed within 60 min showing favorable results across multiple clinical domains. The relationship between surgical duration and outcomes extends beyond infection risk reduction, encompassing various perioperative advantages that can improve the patient experience. Efficient surgery allows for the use of shorter-acting anesthetics, enabling patients to regain complete visceral and extremity control within two hours postoperatively, potentially reducing complications such as urinary retention that can occur with extended procedures [48]. The physiological benefits of abbreviated surgical time include decreased anesthetic intensity requirements, reduced medical complications, lower sedation needs, improved postoperative patient vigor, and simplified fluid and electrolyte management for anesthesiologists [49]. Literature across both hip and knee replacement studies shows lower complication rates associated with shorter surgical times, with systematic reviews indicating that procedures extending beyond 90 min carry increased risks of infection, thromboembolic complications, and other postoperative morbidities [50]. The mechanisms underlying these improved outcomes include reduced tissue exposure time, minimized inflammatory response activation, decreased anesthetic burden on organ systems, and preserved physiological reserves that support recovery capacity [51]. Contemporary data suggests that surgical teams achieving consistent sub-60 min operative times create a favorable environment for enhanced recovery protocols, where patients benefit from reduced systemic stress, faster emergence from anesthesia, earlier mobilization, and decreased overall physiological perturbation [52]. This evidence supports efficient surgical technique as a valuable component of comprehensive perioperative optimization that can contribute to improvements in patient safety, comfort, and functional recovery trajectories.

### 3.6. Optimized Component Sizing: Fine Increment Femoral Components and Asymmetric Tibial Designs

Component sizing optimization through fine increment femoral components and asymmetric tibial designs represents a transformative game changer that addresses the fundamental anatomical variability across patient populations while maximizing bone-implant interface optimization. Contemporary evidence demonstrates that femoral component systems offering 2 mm incremental sizing with multiple medial-lateral options per anteroposterior size achieve superior component-to-bone fit compared to traditional designs with larger sizing gaps [53,54]. The clinical significance of this precision becomes evident when considering that an overhang of ≥3 mm approximately doubles the incidence of clinically important knee pain at two years post-TKA, while decreased posterior condylar offset of just 2 mm reduces postoperative flexion by 12.2° [55].

Advanced implant designs with extensive sizing matrices demonstrate measurable clinical advantages across multiple outcome domains. Systems providing 9–12 anteroposterior sizes with multiple medial-lateral offerings achieve optimal coverage rates of 76–78% compared to only 61% for limited-size systems, with anatomical designs showing particularly superior medial-posterior coverage (48% optimal fit versus 0–4% for symmetrical designs) and enhanced medial-lateral coverage (42% versus 32–38%) [56]. The physiological benefits of optimal component fit extend beyond simple coverage metrics, with properly sized components reducing femoral notching (one case versus three cases), preserving greater bone stock, and achieving superior posterior condylar offset restoration (5.89 mm versus 1.31 mm mean increase) [56]. These technical improvements translate directly to reduced periprosthetic fracture risk through minimized anterior femoral notching, decreased soft tissue impingement from reduced component overhang, and enhanced postoperative range of motion through optimized posterior condylar offset maintenance.

Asymmetric tibial component designs represent an equally significant advancement that addresses the inherent anatomical asymmetry of the tibial plateau, with systematic reviews and meta-analyses demonstrating that asymmetric tibial components achieve significantly superior tibial coverage compared to symmetric designs [57]. This advantage persists regardless of positioning strategy, whether components are aligned to the medial third of the tubercle or positioned for maximum coverage, with asymmetric designs consistently providing better coverage rates in both actual and simulated TKA procedures [58]. The clinical implications of improved tibial coverage extend beyond simple bone preservation, as optimal tibial component positioning reduces stress concentration at the bone-implant interface, minimizes the risk of component subsidence, and enhances long-term fixation stability while preserving maximal bone stock for potential future revision procedures [54,59].

The integration of fine increment femoral sizing with asymmetric tibial designs creates a comprehensive approach to component optimization that respects individual anatomical variability while maximizing surgical precision. This evidence-based sizing strategy enables surgeons to achieve optimal component fit across diverse patient populations, particularly addressing the morphological differences observed across ethnic groups, where traditional sizing systems may inadequately accommodate distal femur aspect ratio variations [60]. The combination of precise femoral sizing and anatomically appropriate tibial design represents a fundamental evolution in implant technology that translates theoretical engineering advantages into measurable clinical benefits, establishing component sizing optimization as an essential element of contemporary TKA that directly impacts patient satisfaction, functional outcomes, and long-term implant survivorship.

### 3.7. Optimized Tourniquet Management

The personalized optimization of tourniquet application represents a significant game changer in TKA. Comprehensive meta-analyses involving over 2800 patients demonstrate that while traditional full-duration tourniquet use provides marginal intraoperative benefits, it significantly increases all complication risk (risk ratio 1.73), elevates postoperative pain scores by 1.25 points on the first day, and extends hospital stays by 0.34 days without meaningful clinical advantages in blood loss management or functional outcomes [61]. Contemporary evidence reveals that personalized tourniquet inflation pressures (PTIP) based on individual systolic blood pressure achieve superior outcomes compared to uniform inflation protocols, with patients experiencing significantly reduced pain scores both at rest and during activity in the early postoperative period, enhanced range of motion, higher PROMs, and reduced thigh complications, including decreased ecchymosis rates and VTE events [62].

The paradigm shift toward optimized tourniquet protocols involves either eliminating tourniquet use or implementing short-duration, low-pressure strategies that preserve surgical visibility while minimizing physiological perturbation. Randomized controlled trials comparing no-tourniquet versus optimized tourniquet techniques (inflation before skin incision and deflation after cementing, with pressure 100 mmHg above systolic blood pressure) demonstrate equivalent outcomes across all measured parameters, including surgical timing, blood loss, pain levels, edema, range of motion, functional scores, and complication rates [63]. When tourniquet use is deemed necessary, short-duration protocols of less than 10 min achieve comparable pain control and functional outcomes while significantly reducing opioid consumption at 24 h postoperatively compared to extended tourniquet times, without compromising hemoglobin levels, creatine kinase markers, or knee society scores [63].

Large-scale prospective studies involving over 5600 patients confirm that while tourniquet use may provide minimal short-term advantages in achieving meaningful clinical improvement at one month (55.4% versus 47.9%), this difference disappears by three and six months, with no sustained benefits in pain scores, physical function, or patient-reported outcomes [64]. The evidence consistently demonstrates that avoiding prolonged, high-pressure tourniquet application eliminates the cascade of complications associated with tissue ischemia and reperfusion injury while maintaining surgical precision and patient safety. This approach particularly benefits patients with hypertension, where optimized tourniquet protocols involving application before prosthesis placement and strategic release timing demonstrate superior short-term outcomes, reduced complications, and improved rehabilitation compared to full-procedure tourniquet use [65].

### 3.8. Medial Congruent Bearings

Medial congruent bearing designs represent an evolving approach in TKA that may benefit patients with high functional demands and active lifestyles. Current evidence suggests that medial congruence, ranging from partial to total congruency in medial pivot designs, can be a suitable choice for TKA patients who engage in activities beyond basic daily living [66]. Literature comparing medial congruent or medial pivot designs to posterior-stabilized implants shows consistent equality or potential superiority across multiple outcome measures, with studies generally not demonstrating inferiority of congruent designs [67]. For patients requiring high walking speeds, ambulation on uneven surfaces, stair navigation, and incline walking, the enhanced stability provided by these articulations may deliver improved outcomes [68]. This clinical potential is reflected in recent American registry data showing increased adoption of medially congruent implants, indicating growing clinical acceptance based on observed benefits. Meta-analyses encompassing over 1400 knees suggest that medial pivot designs can achieve better WOMAC scores compared to posterior-stabilized implants, with potentially lower overall complication rates and reduced local complication incidence [69]. The functional advantages may extend beyond basic outcome measures, with medially stabilized designs demonstrating higher patient-reported outcome measures and a wider range of motion [70]. Biomechanical studies show reduced anteroposterior displacement, particularly at 45° of flexion, where medially stabilized designs demonstrate only 3.6 mm displacement versus 16.5 mm in posterior-stabilized implants [71]. Functional outcome data indicates faster and more frequent return to sports activity in medially stabilized patients compared to posterior-stabilized groups [72]. Registry data supports this trend, with posterior-stabilized bearings showing higher hazard ratios for all-cause revision and infection-related revision compared to pivot bearing designs [35]. This evidence suggests that medial congruent designs may be a beneficial choice for active patients seeking to optimize their functional potential, potentially providing enhanced stability, improved range of motion, reduced complications, and better long-term survivorship compared to traditional posteriorly stabilized alternatives.

## 4. Postoperative Game Changers

### 4.1. Early Mobilization

Early mobilization within 12 h following TKA has emerged as a critical component of modern perioperative care, significantly altering patient recovery patterns and clinical outcomes. Comprehensive research validates this proactive mobilization strategy, revealing that patients who achieve ambulation during the initial 12 h postoperative period demonstrate substantial physiological and functional improvements that extend well beyond expedited hospital discharge [73]. The therapeutic benefits encompass multiple clinical domains, including shortened hospital stays, improved knee flexion mobility, reduced pain scores, decreased healthcare expenditures, lower rates of thromboembolic complications and pulmonary morbidity, reduced opioid requirements, enhanced rehabilitation performance, and increased rates of direct home discharge compared to institutional care facilities [74]. Nevertheless, the successful implementation of early mobilization necessitates comprehensive Enhanced Recovery After Surgery protocols that systematically address all aspects of perioperative management, including thorough preoperative patient optimization, multimodal analgesic strategies, effective hemostatic control, and adequate infrastructure to support physical therapy interventions on the operative day [75]. The operational complexity requires the elimination of conventional impediments such as excessive drainage systems, indwelling urinary catheters, and preventive measures against urinary retention that have traditionally hindered early mobilization initiatives [76]. Large-scale clinical studies demonstrate quantifiable benefits for ultra-early mobilization, with patients receiving physical therapy within 12 h achieving reduced hospital duration compared to those mobilized between 12 and 24 h, with even greater advantages relative to delayed mobilization at 24–48 h [77]. Although functional assessment scores at three months demonstrate equivalent outcomes between ultra-early and early mobilization cohorts, indicating that both strategies achieve superior long-term results, the immediate perioperative benefits of ultra-early mobilization contribute to enhanced patient satisfaction, reduced healthcare resource consumption, and optimized recovery efficiency [78]. Successful implementation of early mobilization protocols demands systematic collaboration among anesthesia providers, nursing staff, rehabilitation specialists, and surgical teams. When properly executed, this approach establishes an accelerated recovery framework that optimizes clinical outcomes and healthcare efficiency while preserving the safety and effectiveness standards fundamental to contemporary joint replacement surgery.

### 4.2. Appropriate Anticoagulation

Strategic anticoagulant selection constitutes a fundamental determinant of TKA success, with hemarthrosis prevention serving as a cornerstone of optimal postoperative recovery. This therapeutic approach is predicated on the principle that knees with minimal postoperative bleeding demonstrate markedly superior recovery patterns compared to those with significant swelling, as excessive anticoagulation beyond aspirin therapy generates articular bleeding and secretions that initiate a detrimental cascade compromising both immediate and extended outcomes.

Current evidence, notably from the 2022 VTE International Consensus Meeting, establishes that heparins, direct oral anticoagulants, and warfarin substantially elevate postoperative complication rates, with wound-related complications serving as the most apparent indicator of excessive bleeding risk [79]. Low-dose aspirin (81–100 mg twice daily) has been validated as the most efficacious and safe thromboprophylactic intervention, receiving endorsement from both the American Academy of Orthopaedic Surgeons for patients with standard thromboembolic risk profiles and the American College of Chest Physicians since 2012. This recommendation is substantiated by Level I-IV evidence confirming that low-dose aspirin achieves optimal equilibrium between thromboembolic protection and hemorrhage risk mitigation.

The clinical ramifications are substantial, with hematoma development ranking among the third through seventh most frequent causes of hospital readmission following arthroplasty procedures [80]. Comparative analyses between direct oral anticoagulants and aspirin demonstrate notable differences in wound complication incidence, with aspirin demonstrating superior outcomes and emphasizing the significant impact of anticoagulant selection on surgical site healing [81,82,83]. The pathophysiology underlying these complications extends beyond simple hemorrhage, as hemarthrosis establishes an inflammatory microenvironment that impairs tissue repair mechanisms, elevates infection susceptibility, delays mobilization protocols, compromises range of motion restoration, and potentially predisposes patients to periprosthetic joint infection through compromised local immune response [84,85]. This evidence framework positions anticoagulant selection not simply as a pharmacological consideration but as a critical surgical outcome modifier, where aspirin selection over more potent alternatives can differentiate between uncomplicated recovery and complex postoperative trajectories characterized by wound complications, hospital readmissions, and suboptimal functional restoration.

### 4.3. Pain and Swelling Control

Effective management of postoperative pain and inflammation during the recovery period following TKA represents a therapeutic intervention that fundamentally influences recovery outcomes and functional restoration. Although pain management may seem intuitively important, the complex pathophysiological responses to surgical trauma involving substantial bone resection and extensive soft tissue manipulation generate inflammatory cascades that, without targeted therapeutic intervention, sustain persistent pain and edema with extensive complications extending beyond patient comfort considerations. Evidence has established a direct relationship between effective inflammation control and postoperative recovery metrics, with pain management efficacy serving as the critical determinant of successful rehabilitation engagement [86]. Patients achieving optimal pain control, as demonstrated by substantially lower visual analog scale scores, exhibit markedly accelerated attainment of physical therapy benchmarks, with rehabilitation exercise performance being directly enhanced through reduced activity-related discomfort [86]. Beyond functional achievements, effective pain and inflammation management results in significantly reduced difficulty with daily activities, decreased gait abnormalities, fewer medication-related adverse effects, and reduced incidence of complications that compromise therapeutic participation [87]. The underlying mechanism reflects the understanding that inadequately controlled pain and inflammation establish a self-perpetuating cycle wherein inflammatory responses impair tissue repair processes, restrict mobility, delay functional recovery, and compromise patient participation in essential rehabilitation protocols [88]. Contemporary pain management in TKA follows a multimodal analgesic approach based on the World Health Organization analgesic ladder, incorporating non-opioid analgesics (acetaminophen, NSAIDs), regional anesthesia techniques, and judicious opioid use when necessary [89]. However, each component carries specific considerations: NSAIDs may increase bleeding risk and compromise kidney clearance, opioids can cause delayed mobilization, constipation, nausea, and addiction, while spinal anesthesia may result in temporary motor and visceral issues affecting early rehabilitation [90]. By employing multimodal approaches, the risk for side effects from each individual component is significantly minimized through reduced dosing requirements, while different analgesic methods work synergistically to provide superior pain control compared to single-agent therapies, making comprehensive multimodal pain management an essential cornerstone of successful TKA outcomes [91].

## 5. Initial Promises Without Established Clinical Advantages

Although the previously outlined interventions demonstrate substantial clinical benefits, several contemporary practices have garnered attention despite lacking convincing evidence for clinical superiority, representing interventions that consume healthcare resources without delivering meaningful patient improvements.

Prehabilitation programs, while conceptually attractive, exhibit limited evidence for transformative outcomes, with current literature indicating that no specific exercise-based rehabilitation modality has demonstrated superiority for typical patients [92]. Although exercise-based interventions outperform minimal intervention controls, home-based rehabilitation appears equally effective as supervised outpatient programs, suggesting that elaborate prehabilitation protocols may not warrant their complexity and associated costs [92].

Alternative surgical approaches beyond the conventional parapatellar incision constitute another area of excessive expectations. Comprehensive meta-analyses demonstrate no clinically meaningful differences between various surgical approaches in patient-reported outcome measures at six months, one year, or two years postoperatively [93]. The midvastus approach actually exhibits increased complication rates compared to both parapatellar and subvastus techniques [93]. The quadriceps-sparing approach, despite theoretical benefits, showed no clinically significant advantages while substantially increasing femoral, tibial, and mechanical axis outliers alongside prolonged surgical and tourniquet durations [93].

Infrapatellar fat pad preservation versus resection illustrates another intervention without improved clinical effect, with meta-analyses of multiple randomized controlled trials encompassing thousands of patients revealing no clinically important differences [94].

Synovectomy represents a potentially detrimental intervention, with pooled randomized controlled trial results demonstrating significantly increased blood loss and hemoglobin reduction without compensatory benefits in pain relief, functional improvement, or range of motion enhancement [95]. Similarly, patellar denervation shows no differences in pain levels, range of motion, reoperation rates, or patient satisfaction compared to preservation techniques [96].

Patient-specific instrumentation, despite theoretical precision advantages, demonstrates no clinical superiority in functional outcomes at any postoperative interval when compared to conventional instrumentation systems [97]. Sensor-embedded polyethylene components represent another technological advancement without clinical benefit, with studies showing no significant differences in patient-reported outcomes or range of motion between sensor-guided and manually balanced techniques at meaningful follow-up periods [98].

Computer-assisted surgical navigation exemplifies the disconnection between technological sophistication and clinical outcomes, with comprehensive meta-analyses revealing no significant differences in patient-reported outcomes or pain measures despite improved component positioning accuracy [99].

## 6. Limitations

This narrative review contains several inherent limitations that require acknowledgment. The analysis primarily integrates existing literature with clinical practice observations and preferences. The narrative methodology, while enabling comprehensive synthesis, lacks the systematic approach and statistical rigor characteristic of formal meta-analyses, potentially affecting the robustness of our findings. Additionally, institutional differences in surgical protocols, patient demographics, and healthcare delivery systems may influence the generalizability of the described outcomes across different practice settings.

## 7. Conclusions

While novel interventions in TKA often generate initial enthusiasm through optimistic reports and theoretical benefits, rigorous evaluation frequently reveals failure to demonstrate sustained clinically meaningful improvements. The interventions identified in this review have progressed far beyond initial promise to demonstrate consistent, measurable, meaningful, and systematically validated improvements in patient outcomes that changed clinical practice. This distinction enables clinicians to prioritize evidence-based practices with proven clinical superiority while avoiding resource allocation to interventions that lack demonstrated efficacy.

## Data Availability

Not applicable.

## References

[1] Oussedik S., Haddad F.S. (2025). Is Shifting the Goal Posts a Game-Changer in Total Knee Arthroplasty?. Bone Jt. J..

[2] Rak D., Klann L., Heinz T., Anderson P., Stratos I., Nedopil A.J., Rudert M. (2023). Influence of Mechanical Alignment on Functional Knee Phenotypes and Clinical Outcomes in Primary TKA: A 1-Year Prospective Analysis. J. Pers. Med..

[3] Connelly J.W., Galea V.P., Rojanasopondist P., Nielsen C.S., Bragdon C.R., Kappel A., Huddleston J.I., Malchau H., Troelsen A. (2020). Which Preoperative Factors Are Associated with Not Attaining Acceptable Levels of Pain and Function After TKA? Findings from an International Multicenter Study. Clin. Orthop. Relat. Res..

[4] Polkowski G.G., Ruh E.L., Barrack T.N., Nunley R.M., Barrack R.L. (2013). Is Pain and Dissatisfaction after TKA Related to Early-Grade Preoperative Osteoarthritis?. Clin. Orthop. Relat. Res..

[5] Leppänen S., Niemeläinen M., Huhtala H., Eskelinen A. (2021). Mild Knee Osteoarthritis Predicts Dissatisfaction after Total Knee Arthroplasty: A Prospective Study of 186 Patients Aged 65 Years or Less with 2-Year Follow-Up. BMC Musculoskelet. Disord..

[6] Meding J.B., Ritter M.A., Faris P.M., Keating E.M., Harris W. (2001). Does the Preoperative Radiographic Degree of Osteoarthritis Correlate to Results in Primary Total Knee Arthroplasty?. J. Arthroplast..

[7] Riis A., Rathleff M.S., Jensen M.B., Simonsen O. (2014). Low Grading of the Severity of Knee Osteoarthritis Pre-Operatively Is Associated with a Lower Functional Level after Total Knee Replacement: A Prospective Cohort Study with 12 Months’ Follow-Up. Bone Jt. J..

[8] Gu A., Malahias M.-A., Selemon N.A., Wei C., Gerhard E.F., Cohen J.S., Fassihi S.C., Stake S., Bernstein S.L., Chen A.Z. (2020). Increased Severity of Anaemia Is Associated with 30-Day Complications Following Total Joint Replacement. Bone Jt. J..

[9] Shohat N., Tarabichi M., Tan T.L., Goswami K., Kheir M., Malkani A.L., Shah R.P., Schwarzkopf R., Parvizi J. (2019). 2019 John Insall Award: Fructosamine Is a Better Glycaemic Marker Compared with Glycated Haemoglobin (HbA1C) in Predicting Adverse Outcomes Following Total Knee Arthroplasty: A Prospective Multicentre Study. Bone Jt. J..

[10] Kim K.Y., Anoushiravani A.A., Chen K.K., Li R., Bosco J.A., Slover J.D., Iorio R. (2019). Perioperative Orthopedic Surgical Home: Optimizing Total Joint Arthroplasty Candidates and Preventing Readmission. J. Arthroplast..

[11] Bernstein D.N., Liu T.C., Winegar A.L., Jackson L.W., Darnutzer J.L., Wulf K.M., Schlitt J.T., Sardan M.A., Bozic K.J. (2018). Evaluation of a Preoperative Optimization Protocol for Primary Hip and Knee Arthroplasty Patients. J. Arthroplast..

[12] Schroer W.C., LeMarr A.R., Mills K., Childress A.L., Morton D.J., Reedy M.E. (2019). 2019 Chitranjan S. Ranawat Award: Elective Joint Arthroplasty Outcomes Improve in Malnourished Patients with Nutritional Intervention: A Prospective Population Analysis Demonstrates a Modifiable Risk Factor. Bone Jt. J..

[13] Liimakka A.P., Farid A.R., Zhu L., Monette P.J., Varady N.H., Lange J.K., Javedan H., Chen A.F. (2025). Perioperative Geriatrician Assessment Is Associated with a Lower Risk of Emergency Department Visits After Total Joint Arthroplasty. J. Bone Jt. Surg. Am..

[14] Morsi E. (2002). Continuous-Flow Cold Therapy after Total Knee Arthroplasty. J. Arthroplast..

[15] Guillot X., Tordi N., Laheurte C., Pazart L., Prati C., Saas P., Wendling D. (2019). Local Ice Cryotherapy Decreases Synovial Interleukin 6, Interleukin 1β, Vascular Endothelial Growth Factor, Prostaglandin-E2, and Nuclear Factor Kappa B P65 in Human Knee Arthritis: A Controlled Study. Arthritis Res. Ther..

[16] de Vries A.J., Aksakal H.K., Brouwer R.W. (2024). Effects of 6 Weeks of Cryotherapy plus Compression Therapy after Total or Unicompartmental Knee Arthroplasty: Protocol for a Single-Centre, Single-Blind Randomised Controlled Trial. BMJ Open.

[17] Hohenauer E., Clarys P., Baeyens J.-P., Clijsen R. (2016). The Effect of Local Cryotherapy on Subjective and Objective Recovery Characteristics Following an Exhaustive Jump Protocol. Open Access J. Sports Med..

[18] Pieri L., Leggieri F., Bartoli D., Ponti M., Caparrini C., Baldini A. (2025). Preoperative Knee Joint Hypothermia Reduces Inflammation and Recovery Time and Increases Range of Motion after Total Knee Arthroplasty: A Randomized Controlled Trial. Knee Surg. Sports Traumatol. Arthrosc..

[19] Gasbjerg K.S., Hägi-Pedersen D., Lunn T.H., Laursen C.C., Holmqvist M., Vinstrup L.Ø., Ammitzboell M., Jakobsen K., Jensen M.S., Pallesen M.J. (2022). Effect of Dexamethasone as an Analgesic Adjuvant to Multimodal Pain Treatment after Total Knee Arthroplasty: Randomised Clinical Trial. BMJ.

[20] Xu B., Ma J., Huang Q., Huang Z.-Y., Zhang S.-Y., Pei F.-X. (2018). Two Doses of Low-Dose Perioperative Dexamethasone Improve the Clinical Outcome after Total Knee Arthroplasty: A Randomized Controlled Study. Knee Surg. Sports Traumatol. Arthrosc..

[21] Chia S.K., Wernecke G.C., Harris I.A., Bohm M.T., Chen D.B., Macdessi S.J. (2013). Peri-Articular Steroid Injection in Total Knee Arthroplasty: A Prospective, Double Blinded, Randomized Controlled Trial. J. Arthroplast..

[22] Lunn T.H., Kristensen B.B., Andersen L.Ø., Husted H., Otte K.S., Gaarn-Larsen L., Kehlet H. (2011). Effect of High-Dose Preoperative Methylprednisolone on Pain and Recovery after Total Knee Arthroplasty: A Randomized, Placebo-Controlled Trial. Br. J. Anaesth..

[23] Yue C., Wei R., Liu Y. (2017). Perioperative Systemic Steroid for Rapid Recovery in Total Knee and Hip Arthroplasty: A Systematic Review and Meta-Analysis of Randomized Trials. J. Orthop. Surg. Res..

[24] Lindberg-Larsen V., Ostrowski S.R., Lindberg-Larsen M., Rovsing M.L., Johansson P.I., Kehlet H. (2017). The Effect of Pre-Operative Methylprednisolone on Early Endothelial Damage after Total Knee Arthroplasty: A Randomised, Double-Blind, Placebo-Controlled Trial. Anaesthesia.

[25] MacDessi S.J., Griffiths-Jones W., Harris I.A., Bellemans J., Chen D.B. (2021). Coronal Plane Alignment of the Knee (CPAK) Classification. Bone Jt. J..

[26] Franceschetti E., Campi S., Giurazza G., Tanzilli A., Gregori P., Laudisio A., Hirschmann M.T., Samuelsson K., Papalia R. (2024). Mechanically Aligned Total Knee Arthroplasty Does Not Yield Uniform Outcomes across All Coronal Plane Alignment of the Knee (CPAK) Phenotypes. Knee Surg. Sports Traumatol. Arthrosc..

[27] Yang H., Park C., Cheon J., Hwang J., Seon J. (2025). Comparison of Outcomes Between Functionally and Mechanically Aligned Total Knee Arthroplasty: Analysis of Parallelism to the Ground and Weight-Bearing Position of the Knee Using Hip-to-Calcaneus Radiographs. J. Pers. Med..

[28] Fillingham Y.A., Darrith B., Calkins T.E., Abdel M.P., Malkani A.L., Schwarzkopf R., Padgett D.E., Culvern C., Sershon R.A., Bini S. (2019). 2019 Mark Coventry Award: A Multicentre Randomized Clinical Trial of Tranexamic Acid in Revision Total Knee Arthroplasty: Does the Dosing Regimen Matter?. Bone Jt. J..

[29] Elmenawi K.A., Mohamed F.A.E., Poilvache H., Prokop L.J., Abdel M.P., Bedard N.A. (2024). Association Between Tranexamic Acid and Decreased Periprosthetic Joint Infection Risk in Patients Undergoing Total Hip and Knee Arthroplasty: A Systematic Review and Meta-Analysis of Over 2 Million Patients. J. Arthroplast..

[30] Zhang Q., Chen Y., Li Y., Liu R., Rai S., Li J., Hong P. (2024). Enhanced Recovery after Surgery in Patients after Hip and Knee Arthroplasty: A Systematic Review and Meta-Analysis. Postgrad. Med. J..

[31] Chen Z., Bains S.S., Sax O.C., Sodhi N., Mont M.A. (2024). Optimal Method of Skin Wound Management for Total Knee Arthroplasty: A Systematic Review and Meta-Analysis. J. Knee Surg..

[32] Ainslie-Garcia M., Anderson L.A., Bloch B.V., Board T.N., Chen A.F., Craigie S., Danker W., Gunja N., Harty J., Hernandez V.H. (2024). International Delphi Study on Wound Closure and Incision Management in Joint Arthroplasty Part 2: Total Hip Arthroplasty. J. Arthroplast..

[33] Romanini E., Zanoli G.A., Ascione T., Balato G., Baldini A., Foglia E., Pellegrini A.V., Verde F., Zaffagnini S. (2024). Barbed Sutures and Skin Adhesives Improve Wound Closure in Hip and Knee Arthroplasty. Knee Surg. Sports Traumatol. Arthrosc..

[34] Tan Z., Tomaszewski J., Chen B.P.-H., Gunja N.J., Etter K. (2024). Use of Interrupted Time-Series Analyses in Evaluating Health Economic Outcomes Following Implementation of Multilayer Water-Tight Wound Closure in a Primary Total Joint Arthroplasty Population. J. Comp. Eff. Res..

[35] American Academy of Orthopaedic Surgeons (AAOS) (2024). 2024 Annual Report.

[36] Australian Orthopaedic Association National Joint Replacement Registry (AOANJRR) (2024). Hip, Knee and Shoulder Arthroplasty: 2024 Annual Report.

[37] Wang K., Sun H., Zhang K., Li S., Wu G., Zhou J., Sun X. (2020). Better Outcomes Are Associated with Cementless Fixation in Primary Total Knee Arthroplasty in Young Patients: A Systematic Review and Meta-Analysis of Randomized Controlled Trials. Medicine.

[38] Chen C., Li R. (2019). Cementless versus Cemented Total Knee Arthroplasty in Young Patients: A Meta-Analysis of Randomized Controlled Trials. J. Orthop. Surg. Res..

[39] Sinicrope B.J., Feher A.W., Bhimani S.J., Smith L.S., Harwin S.F., Yakkanti M.R., Malkani A.L. (2019). Increased Survivorship of Cementless versus Cemented TKA in the Morbidly Obese. A Minimum 5-Year Follow-Up. J. Arthroplast..

[40] Goh G.S., Fillingham Y.A., Sutton R.M., Small I., Courtney P.M., Hozack W.J. (2022). Cemented Versus Cementless Total Knee Arthroplasty in Obese Patients With Body Mass Index ≥35 Kg/M2: A Contemporary Analysis of 812 Patients. J. Arthroplast..

[41] Dembek D.J., Bicket M.C. (2023). Advances in the Management of Persistent Pain after Total Knee Arthroplasty. Curr. Opin. Anaesthesiol..

[42] Zhao C., Liao Q., Yang D., Yang M., Xu P. (2024). Advances in Perioperative Pain Management for Total Knee Arthroplasty: A Review of Multimodal Analgesic Approaches. J. Orthop. Surg. Res..

[43] Lavand’homme P.M., Kehlet H., Rawal N., Joshi G.P. (2022). Pain Management after Total Knee Arthroplasty. Eur. J. Anaesthesiol..

[44] Sikachi R.R., Campbell B., Kassin E., Scuderi G.R., Marino J. (2023). Analgesic Trends in the Management of Pain Following Total Knee Arthroplasty: A Comparison of Peri-Articular Infiltration, Adductor Canal Block, and Adjuvant Treatment for Posterior Knee Pain. Orthop. Clin. N. Am..

[45] Tsukada S., Kurosaka K., Nishino M., Hirasawa N. (2018). Cutaneous Hypesthesia and Kneeling Ability After Total Knee Arthroplasty: A Randomized Controlled Trial Comparing Anterolateral and Anteromedial Skin Incision. J. Arthroplast..

[46] Maniar R.N., Singhi T., Nanivadekar A., Maniar P.R., Singh J. (2017). A Prospective Randomized Study in 20 Patients Undergoing Bilateral TKA Comparing Midline Incision to Anterolateral Incision. J. Orthop. Traumatol..

[47] Dhillon M.S., Jindal K., Shetty V.D., Kumar P., Rajnish R.K. (2021). Autonomic Denervation Dermatitis: A Relatively Undocumented ’ADD’itional Complication of Total Knee Replacements and Other Surgeries Around the Knee. Indian J. Orthop..

[48] Duchman K.R., Pugely A.J., Martin C.T., Gao Y., Bedard N.A., Callaghan J.J. (2017). Operative Time Affects Short-Term Complications in Total Joint Arthroplasty. J. Arthroplast..

[49] Schroer W.C., Calvert G.T., Diesfeld P.J., Reedy M.E., LeMarr A.R. (2008). Effects of Increased Surgical Volume on Total Knee Arthroplasty Complications. J. Arthroplast..

[50] Pulido L., Ghanem E., Joshi A., Purtill J.J., Parvizi J. (2008). Periprosthetic Joint Infection: The Incidence, Timing, and Predisposing Factors. Clin. Orthop. Relat. Res..

[51] Frisch N.B., Darrith B., Hansen D.C., Wells A., Sanders S., Berger R.A. (2018). Single-Dose Lidocaine Spinal Anesthesia in Hip and Knee Arthroplasty. Arthroplast. Today.

[52] Pugely A.J., Martin C.T., Gao Y., Mendoza-Lattes S., Callaghan J.J. (2013). Differences in Short-Term Complications between Spinal and General Anesthesia for Primary Total Knee Arthroplasty. J. Bone Jt. Surg. Am..

[53] Cheng F.B., Ji X.F., Lai Y., Feng J.C., Zheng W.X., Sun Y.F., Fu Y.W., Li Y.Q. (2009). Three Dimensional Morphometry of the Knee to Design the Total Knee Arthroplasty for Chinese Population. Knee.

[54] Dai Y., Scuderi G.R., Bischoff J.E., Bertin K., Tarabichi S., Rajgopal A. (2014). Anatomic Tibial Component Design Can Increase Tibial Coverage and Rotational Alignment Accuracy: A Comparison of Six Contemporary Designs. Knee Surg. Sports Traumatol. Arthrosc..

[55] Hitt K., Shurman J.R., Greene K., McCarthy J., Moskal J., Hoeman T., Mont M.A. (2003). Anthropometric Measurements of the Human Knee: Correlation to the Sizing of Current Knee Arthroplasty Systems. J. Bone Jt. Surg. Am..

[56] Maciąg B.M., Stolarczyk A., Maciąg G.J., Dorocińska M., Stępiński P., Szymczak J., Świercz M., Żarnovsky K., Łapiński M., Stolarczyk M. (2021). Does the Anatomic Design of Total Knee Prosthesis Allow for a Better Component Fit than Its Nonanatomic Predecessor? A Matched Cohort Study. Arthroplast. Today.

[57] Mahoney O.M., Kinsey T. (2010). Overhang of the Femoral Component in Total Knee Arthroplasty: Risk Factors and Clinical Consequences. J. Bone Jt. Surg. Am..

[58] Hsu R.W., Himeno S., Coventry M.B., Chao E.Y. (1990). Normal Axial Alignment of the Lower Extremity and Load-Bearing Distribution at the Knee. Clin. Orthop. Relat. Res..

[59] Zhang Z., Zhang T., Zhang L., Chen Z., Zhao H., Kuang J., Ou L. (2024). Comparison of the Coverage and Rotation of Asymmetrical and Symmetrical Tibial Components: A Systematic Review and Meta-Analysis. BMC Musculoskelet. Disord..

[60] Shekhar A., Chandra Krishna C., Patil S., Tapasvi S. (2020). Does Increased Femoral Component Size Options Reduce Anterior Femoral Notching in Total Knee Replacement?. J. Clin. Orthop. Trauma.

[61] Cai D.F., Fan Q.H., Zhong H.H., Peng S., Song H. (2019). The Effects of Tourniquet Use on Blood Loss in Primary Total Knee Arthroplasty for Patients with Osteoarthritis: A Meta-Analysis. J. Orthop. Surg. Res..

[62] Pavão D.M., Pires eAlbuquerque R.S., de Faria J.L.R., Sampaio Y.D., de Sousa E.B., Fogagnolo F. (2023). Optimized Tourniquet Use in Primary Total Knee Arthroplasty: A Comparative, Prospective, and Randomized Study. J. Arthroplast..

[63] Zak S.G., Yeroushalmi D., Long W.J., Meftah M., Schnaser E., Schwarzkopf R. (2021). Does the Use of a Tourniquet Influence Outcomes in Total Knee Arthroplasty: A Randomized Controlled Trial. J. Arthroplast..

[64] Ahmed I., Chawla A., Underwood M., Price A.J., Metcalfe A., Hutchinson C.E., Warwick J., Seers K., Parsons H., Wall P.D.H. (2021). Time to Reconsider the Routine Use of Tourniquets in Total Knee Arthroplasty Surgery. Bone Jt. J..

[65] Ahmed I., Chawla A., Underwood M., Price A.J., Metcalfe A., Hutchinson C., Warwick J., Seers K., Parsons H., Wall P.D. (2020). Tourniquet Use for Knee Replacement Surgery. Cochrane Database Syst. Rev..

[66] Shi W., Jiang Y., Wang Y., Zhao X., Yu T., Li T. (2022). Medial Pivot Prosthesis Has a Better Functional Score and Lower Complication Rate than Posterior-Stabilized Prosthesis: A Systematic Review and Meta-Analysis. J. Orthop. Surg. Res..

[67] Liu X., Liu Y., Li B., Wang L., Wang Y., Liu J. (2022). Comparison of the Clinical and Patient-Reported Outcomes between Medial Stabilized and Posterior Stabilized Total Knee Arthroplasty: A Systematic Review and Meta-Analysis. Knee.

[68] Obada B., Iliescu M.G., Costea D.O., Petcu L., Popescu A.I. (2025). Comparative Study of Outcomes with Total Knee Arthroplasty: Medial Pivot Prosthesis vs Posterior Stabilized Implant. Prospective Randomized Control. Int. Orthop. (SICOT).

[69] Elbardesy H., Salamah H.M., McLeod A., Thada P.K., Mohammed E.R., Hanifa F.A., Roshdy M., Guerin S. (2021). Medial Pivot versus (Cam Post) Posterior Stabilised Total Knee Arthroplasty, Systematic Review and Meta-Analysis of 3837 Knees. Acta Orthop. Belg..

[70] Scott D.F., Gray C.G. (2022). Outcomes Are Better With a Medial-Stabilized vs a Posterior-Stabilized Total Knee Implanted With Kinematic Alignment. J. Arthroplast..

[71] Scott D.F., Hellie A.A. (2022). Mid-Flexion, Anteroposterior Stability of Total Knee Replacement Implanted with Kinematic Alignment. J. Bone Jt. Surg..

[72] Kendall J., Pelt C.E., Imlay B., Yep P., Mullen K., Kagan R. (2022). Revision Risk for Total Knee Arthroplasty Polyethylene Designs in Patients 65 Years of Age or Older: An Analysis from the American Joint Replacement Registry. J. Bone Jt. Surg. Am..

[73] Alsiri N., Alshatti S.A., Al-Saffar M., Bhatia R.S., Fairouz F., Palmer S. (2025). EMMATKA Trial: The Effects of Mobilization with Movement Following Total Knee Arthroplasty in Women: A Single-Blind Randomized Controlled Trial. J. Orthop. Surg. Res..

[74] Ripoll S-Melchor J., Aldecoa C.S., Fern Índez-Garc A.R., Varela-Dur Ín M., Aracil-Escoda N., Garc A-Rodr Guez D., Cabezudo-de-la-Muela L., Hormaechea-Bolado L.A., Nacarino-Alcorta B., Hoffmann R. (2023). Early Mobilization after Total Hip or Knee Arthroplasty: A Substudy of the POWER.2 Study. Braz. J. Anesthesiol..

[75] Lei Y.-T., Xie J.-W., Huang Q., Huang W., Pei F.-X. (2021). Benefits of Early Ambulation within 24 h after Total Knee Arthroplasty: A Multicenter Retrospective Cohort Study in China. Mil. Med. Res..

[76] Thwin L., Chee B.R.K., Yap Y.M., Tan K.G. (2024). Total Knee Arthroplasty: Does Ultra-Early Physical Therapy Improve Functional Outcomes and Reduce Length of Stay? A Retrospective Cohort Study. J. Orthop. Surg. Res..

[77] Bohl D.D., Li J., Calkins T.E., Darrith B., Edmiston T.A., Nam D., Gerlinger T.L., Levine B.R., Della Valle C.J. (2019). Physical Therapy on Postoperative Day Zero Following Total Knee Arthroplasty: A Randomized, Controlled Trial of 394 Patients. J. Arthroplast..

[78] Sarpong N.O., Boddapati V., Herndon C.L., Shah R.P., Cooper H.J., Geller J.A. (2019). Trends in Length of Stay and 30-Day Complications After Total Knee Arthroplasty: An Analysis From 2006 to 2016. J. Arthroplast..

[79] Mont M.A., Abdeen A., Abdel M.P., Al Mutani M.N., Amin M.S., Arish A., Azboy I., Baker C.M., Baldini A., Bengoa F. (2022). Recommendations from the ICM-VTE: Hip & Knee. J. Bone Jt. Surg..

[80] Mirghaderi P., Pahlevan-Fallahy M.-T., Rahimzadeh P., Habibi M.A., Pourjoula F., Azarboo A., Moharrami A. (2024). Low-versus High-Dose Aspirin for Venous Thromboembolic Prophylaxis after Total Joint Arthroplasty: A Systematic Review and Meta-Analysis. J. Orthop. Surg. Res..

[81] Warren J.A., Sundaram K., Anis H.K., Kamath A.F., Higuera C.A., Piuzzi N.S. (2020). Have Venous Thromboembolism Rates Decreased in Total Hip and Knee Arthroplasty?. J. Arthroplast..

[82] Migliorini F., Maffulli N., Velaj E., Bell A., Kämmer D., Eschweiler J., Hofmann U.K. (2024). Antithrombotic Prophylaxis Following Total Knee Arthroplasty: A Level I Bayesian Network Meta-Analysis. Eur. J. Orthop. Surg. Traumatol..

[83] Jiang W., Yan Y., Huang T., Lin Z., Yang X., Luo Z., Ye L. (2024). Efficacy and Safety of Aspirin in Venous Thromboembolism Prevention after Total Hip Arthroplasty, Total Knee Arthroplasty or Fracture. Vasa.

[84] Guo X., Zheng S., Zhi Y. (2024). Comment on “The Role of Aspirin versus Low-Molecular-Weight Heparin for Venous Thromboembolism Prophylaxis after Total Knee Arthroplasty: A Meta-Analysis of Randomized Controlled Trials”. Int. J. Surg..

[85] Ding K., Yan W., Zhang Y., Li J., Li C., Liang C. (2024). The Safety and Efficacy of NOACs versus LMWH for Thromboprophylaxis after THA or TKA: A Systemic Review and Meta-Analysis. Asian J. Surg..

[86] Lamplot J.D., Wagner E.R., Manning D.W. (2014). Multimodal Pain Management in Total Knee Arthroplasty: A Prospective Randomized Controlled Trial. J. Arthroplast..

[87] Qiu H., Yu L., Wang Q., Liu Z., Li L. (2024). Clinical Efficacy, Analgesic Efficacy, and Effects of Cocktail Analgesic Regimens in Patients Undergoing Total Knee Arthroplasty: A Systematic Review and Meta-Analysis. Altern. Ther. Health Med..

[88] Jiang J., Teng Y., Fan Z., Khan M.S., Cui Z., Xia Y. (2013). The Efficacy of Periarticular Multimodal Drug Injection for Postoperative Pain Management in Total Knee or Hip Arthroplasty. J. Arthroplast..

[89] Anekar A.A., Hendrix J.M., Cascella M. (2025). WHO Analgesic Ladder. StatPearls.

[90] Edwards I.R., Aronson J.K. (2000). Adverse Drug Reactions: Definitions, Diagnosis, and Management. Lancet.

[91] Schwenk E.S., Mariano E.R. (2018). Designing the Ideal Perioperative Pain Management Plan Starts with Multimodal Analgesia. Korean J. Anesthesiol..

[92] Bandholm T., Wainwright T.W., Kehlet H. (2018). Rehabilitation Strategies for Optimisation of Functional Recovery after Major Joint Replacement. J. Exp. Orthop..

[93] Bouché P.-A., Corsia S., Nizard R., Resche-Rigon M. (2021). Comparative Efficacy of the Different Surgical Approaches in Total Knee Arthroplasty: A Systematic-Review and Network Meta-Analysis. J. Arthroplast..

[94] Walker H., Rao A., Tsimiklis J., Smitham P. (2024). Are Short Term Outcomes Superior Following Total Knee Arthroplasty When Infra-Patellar Fat Pad Is Resected? A Systematic Review and Meta-Analysis. ANZ J. Surg..

[95] Liu P., Lu F., Chen J., Xia Z., Yu H., Zhang Q., Wang W., Guo W. (2019). Should Synovectomy Be Performed in Primary Total Knee Arthroplasty for Osteoarthritis? A Meta-Analysis of Randomized Controlled Trials. J. Orthop. Surg. Res..

[96] Liu L., Li J., Wang Y., Li X., Han P., Li X. (2024). Different Modalities of Patellar Management in Primary Total Knee Arthroplasty: A Bayesian Network Meta-Analysis of Randomized Controlled Trials. J. Orthop. Surg. Res..

[97] Burnett R., Barrack R. (2013). Computer-Assisted Total Knee Arthroplasty Is Currently of No Proven Clinical Benefit: A Systematic Review. Clin. Orthop. Relat. Res..

[98] Sava M.-P., Hara H., Alexandra L., Hügli R.W., Hirschmann M.T. (2023). Verasense Sensor-Assisted Total Knee Arthroplasty Showed No Difference in Range of Motion, Reoperation Rate or Functional Outcomes When Compared to Manually Balanced Total Knee Arthroplasty: A Systematic Review. Knee Surg. Sports Traumatol. Arthrosc..

[99] Namireddy S.R., Gill S.S., Yaqub Y., Ramkumar P. (2024). Computerized Versus Traditional Approaches for Total Knee Arthroplasty: A Quantitative Analysis of Knee Society Score and Western Ontario and McMaster Universities Osteoarthritis Index. Orthop. Surg..

